# The Effect of Synbiotics and Probiotics on Ochratoxin Concentrations in Blood and Tissues, Health Status, and Gastrointestinal Function in Turkeys Fed Diets Contaminated with Ochratoxin A

**DOI:** 10.3390/ani14203024

**Published:** 2024-10-18

**Authors:** Magdalena Mazur-Kuśnirek, Krzysztof Lipiński, Zofia Antoszkiewicz, Katarzyna Śliżewska

**Affiliations:** 1 Department of Animal Nutrition, Feed Science and Cattle Breeding, University of Warmia and Mazury in Olsztyn, 10-718 Olsztyn, Poland; krzysztof.lipinski@uwm.edu.pl (K.L.); zofia.antoszkiewicz@uwm.edu.pl (Z.A.); 2 Institute of Fermentation Technology and Microbiology, Department of Biotechnology and Food Sciences, Lodz University of Technology, 90-924 Łódź, Poland; katarzyna.slizewska@p.lodz.pl

**Keywords:** gastrointestinal tract, health indicators, mycotoxins, ochratoxin A, poultry, probiotic, synbiotic

## Abstract

The aim of this study was to evaluate carcass quality and analyze gastrointestinal functional status, ochratoxin A (OTA) accumulation in tissues and organs, and the health status of turkeys fed diets contaminated with OTA and supplemented with synbiotic preparations in comparison with commercial probiotic feed additives. The research involved 120 female BIG 6 turkeys, divided into six treatment groups (five replicates, four birds per replicate). Wheat naturally contaminated with OTA (662.03 μg/kg) was used in turkey diets. Turkeys in group 1 received an OTA-contaminated diet without additives. Groups 2 and 3 received 0.4 g/kg of probiotic preparation BioPlus 2B or Cylactin. Groups 4, 5, and 6 received 0.5 g/kg of synbiotics S1, S2, or S3. The following parameters were monitored: growth performance, carcass quality, gastrointestinal tract structure and digesta pH, health status, and concentrations of OTA in the blood and tissues of turkeys. The study found no significant differences in the growth performance and carcass quality of turkey. However, the introduction of probiotics or synbiotics into OTA-contaminated feed mixtures resulted in a reduced pH of the digesta in certain sections of the turkey digestive tract (*p* < 0.05). Additionally, the tested synbiotic additives significantly reduced liver weight in turkeys at weeks 6 and 15 (*p* < 0.05). The addition of probiotic and synbiotic preparations based on lactic acid bacteria strains, inulin, and *S. cerevisiae* yeasts to OTA-contaminated diets in commercial turkey farming may improve health status (*p* < 0.05) and reduce mycotoxin accumulation in organs and tissues of poultry (*p* < 0.05).

## Simple Summary

Mycotoxins are secondary metabolites of molds, primarily Penicillium, Fusarium, and Aspergillus species, that may be present in food and feed. Reports indicate that up to 25% of the world’s cereal crops could be contaminated with these fungal toxins. Synbiotics, products that synergistically combine probiotics and prebiotics, can reduce harmful metabolites in the gastrointestinal tract by metabolizing or adsorbing toxic substances, including mycotoxins. In the present study, turkeys were fed diets contaminated with ochratoxin A and supplemented with probiotic or synbiotic preparations. The addition of probiotic and synbiotic preparations based on lactic acid bacteria strains, inulin, and *Saccharomyces cerevisiae* yeast to ochratoxin A-contaminated diets in commercial turkey farming may improve health status and reduce mycotoxin accumulation in poultry organs and tissues.

## 1. Introduction

Mycotoxins are secondary metabolites of molds, primarily *Penicillium* spp., *Fusarium* spp., and *Aspergillus* spp., that may be present in food and feed [1]. It is estimated that more than 25% of the world’s cereal crops may be contaminated with fungal toxins [2]. More than 500 of the mycotoxins identified to date are considered to be harmful [3]. They demonstrate teratogenic, mutagenic, carcinogenic, and immunosuppressive properties in animals and humans [4,5]. Among fungal toxins, ochratoxins appear to be particularly dangerous to birds [6]. Ochratoxin A (OTA) concentrations should not exceed 0.1 mg/kg in compound and supplemental feeds for poultry, and 0.25 mg/kg in cereals and cereal products [7]. Feedstuffs of adequate quality are used as ingredients in livestock diets, and therefore the maximum permitted levels of mycotoxins are rarely exceeded. However, the findings from numerous studies indicate that even relatively low levels of these substances in the diets of young animals can impair their immune function, cause damage to the liver and other organs, reduce feed consumption, increase mortality, and decrease performance [8,9,10]. 

Probiotics and prebiotics can be used to biologically detoxify mycotoxins that are present in feed [11,12,13]. Probiotics are defined as “live microorganisms that confer health benefits to the host when administered in adequate quantities”. Probiotic preparations usually contain single bacterial strains or several different bacterial cultures [14,15]. Probiotic microorganisms are able to reduce the amount of harmful metabolites in the gastrointestinal tract through metabolism or adsorption of toxic substances, including mycotoxins [16,17]. In addition, probiotics exert a beneficial influence by increasing the production of enzymes, vitamins, and antibacterial substances. Another mode of their action is to improve the resistance of the intestinal mucosa to pathogen colonization and to stimulate natural defense mechanisms, resulting in positive health effects [18,19]. Prebiotics are not digested by the host enzymes and are fermented in the gastrointestinal tract, stimulating the proliferation of beneficial bacteria [20]. Synbiotics, i.e., products that combine synergistically acting probiotics and prebiotics, are responsible for maintaining intestinal homeostasis and beneficially affecting animal health by improving the survival of the gut microbiota [21,22]. The stimulatory effect of synbiotics on growth performance and meat quality in poultry raised under standard conditions and in pathogen-infected flocks has been widely documented [23,24,25,26,27,28]. 

In the available literature, there are very few or no results regarding the negative impact of OTA on the health and functional status of the digestive tract, as well as the accumulation of toxins in the organs and tissues of turkeys. Moreover, the effects of synbiotics containing inulin, *Saccharomyces cerevisiae* yeast, and lactic acid bacteria on turkeys exposed to OTA in feed mixtures have not yet been studied.

The research hypothesis postulates that synbiotic supplements can improve carcass quality, gastrointestinal function, and the health status of turkeys exposed to OTA in feed. The aim of this study was to evaluate carcass quality and analyze gastrointestinal functional status, OTA accumulation in tissues and organs, and the health status of turkeys fed diets contaminated with OTA and supplemented with synbiotic preparations in comparison with commercial probiotic feed additives.

## 2. Materials and Methods

### 2.1. Animals, Experimental Design, and Diets

The experimental materials comprised 120 one-day-old, female BIG 6 turkeys (6 treatments, 5 replicates, 4 birds per replicate). The experiment lasted for 15 weeks. The research was conducted on a poultry research farm belonging to the University of Warmia and Mazury in northeastern Poland. The birds were kept on litter, under standard environmental conditions. The following lighting schedule was implemented: 24 h of light (intensity of 100 lx) for the first 72 h, followed by 18 h of light per day until day 14, and then 16 h of light per day until the end of the experiment. Light intensity was reduced to 5 lx from days 3 to 7 and gradually increased to 15 lx starting in week 5. All turkeys were fed standard mash complete diets whose nutritional value was consistent with the nutrient requirements of fast-growing birds (Table 1). The turkeys had unrestricted access to both feed and drinking water.

Wheat naturally contaminated with OTA (662.03 μg/kg) was used as an ingredient of turkey diets. The OTA content of diets increased with increasing inclusion levels of wheat, from 198.6 μg/kg (starter 1 diet) to 462.0 μg/kg (finisher diet). Turkeys from the first group (OTA) received a diet contaminated with OTA, without additives. Birds from groups 2 and 3 were fed contaminated diets supplemented with probiotics, BioPlus 2B and Cylactin, respectively, at 0.4 g/kg of feed. Birds from groups 4, 5, and 6 were administered different synbiotic preparations (S1, S2, and S3) at 0.5 g/kg of feed.

Synbiotic preparations (1, 2, and 3) were designed at the Institute of Fermentation Technology and Microbiology, Lodz University of Technology, Poland. The final formulations were elaborated by the Department of Biotechnology and Food Microbiology, Poznan University of Life Sciences. Each synbiotic contained 2.0 × 10^9^ CFU/g of *Lactobacillus* spp., 2.0 × 10^7^ CFU/g of *Saccharomyces cerevisiae* yeast, and 2% inulin as a prebiotic. The microbial content was identical across all three synbiotics (1, 2, and 3). The experimental design is presented in Table 2. 

During the experiment, the body weight of the turkeys and the amount of feed consumed were monitored weekly, and the data were used to calculate the feed conversion ratio (FCR).

### 2.2. Sample Collection and Laboratory Analyses

Feed samples were assessed for their crude protein (CP), ether extract (EE), and crude fiber (CF) content using standard methods [29]. The value of apparent metabolizable energy and the concentrations of minerals and amino acids in turkey diets were determined according to the *Nutrient Requirements of Poultry* [30].

At 6 and 15 weeks of age, 10 turkeys per group were humanely slaughtered via cervical dislocation, and the structure and function of the gastrointestinal tract were evaluated. Their gastrointestinal tracts were removed, and the following segments were separated and weighed: crop, gizzard, proventriculus, small intestine, and ceca. The small intestine and ceca were measured. Each organ was weighed with and without the contents. The carcass dressing percentage was determined. Breast muscles, abdominal fat, heart, kidneys, and liver were harvested and weighed. pH was measured immediately after slaughter, with a pH meter in digesta samples collected from the crop, gizzard, small intestine, and ceca. The samples of meat, tissues, digesta and feces were stored at a temperature of −80 °C until analysis.

The OTA levels in wheat grain, diets, meat, tissues, digesta, and feces of turkeys were analyzed by immunoaffinity column clean-up and high-performance liquid chromatography (HPLC) fluorescence detection in accordance with Polish Standard PN-EN 16007-2012-U [31]. The concentrations of the extracts were measured by HPLC (LC–20AD, Shimadzu, Japan). The HPLC apparatus consisted of a Jupiter 5u C18 300A chromatography column (Phenomenex), 250 × 4.60 mm; column operating temperature of 24 °C; mobile phase comprising acetonitrile–methanol–aqueous acetic acid (35; 35; 30 *v*/*v*/*v*); flow rate of 0.5 mL/min; injection volume of 100 μL; and a fluorescence detector with λex = 333 nm and λem = 467 nm. The qualitative analysis of the obtained chromatograms involved comparing the retention time of the mycotoxin in the standard solution with the retention time of the corresponding analyte in the actual sample. For quantitative analysis, the mycotoxin concentration in the sample was determined using the standard curve, followed by necessary calculations. Each sample was analyzed in duplicate.

Blood samples were collected from the wing veins of ten birds in each group at 3, 8, and 15 weeks of age. The serum obtained by centrifugation (1500 rpm, 15 min, 4 °C) was pipetted into separate tubes, frozen at −80 °C, and stored until further analysis. Serum OTA levels were determined according to the method of Beker and Radic [32]. Mycotoxin analyses were performed using HPLC with fluorescence detection. The HPLC system was Hewlett Packard 1046A with λex = 333 nm/λem = 477 nm, injection volume of 100 µL, mobile phase comprising acetonitrile–methanol–water–aqueous acetic acid (35:35:29:1); and Symmetry Waters C18 column, 5 µm, 150 mm. The chromatographs were integrated and quantified using Chromax 10 software (Pol-Lab, Warsaw, Poland).

Serum total protein was determined using the spectrophotometric method [33], while γ-globulin levels were determined through precipitation [34]. The turbidimetric assay was employed to determine lysozyme (muramidase) levels [35], and ceruloplasmin activity was measured spectrophotometrically [36].

### 2.3. Statistical Analysis

The results were processed statistically by one-way analysis of variance (ANOVA) and Tukey’s test. Arithmetic means (x) and the standard error of the mean (SEM) were calculated. Significance levels were set at *p* < 0.05, and x, y—*p* > 0.05–*p* < 0.1 were regarded as tendencies. The calculations were performed with the use of STATISTICA 12.0 software.

## 3. Results

### 3.1. Growth Performance

The growth performance of turkeys is presented in Table 3. In week 6 of the experiment, dietary supplementation with synbiotics S1 and S3 increased the body weight of birds (by approx. 8%) relative to the remaining groups (*p* < 0.05). The values of FCR were similar in all groups. No significant differences in the growth performance of turkeys were observed in week 15 of the study.

### 3.2. Gastrointestinal Parameters

Gastrointestinal parameters in 6-week-old turkeys are presented in Table 4. The pH of crop digesta was higher (*p* ≤ 0.01) in birds fed diets supplemented with synbiotic S2. The tested feed additives had no effect on the weight of the crop, proventriculus, or gizzard in turkeys fed OTA-contaminated diets. Birds administered the probiotic preparation Cylactin were characterized by a lower pH of proventriculus digesta than those receiving BioPlus 2B and synbiotics S2 and S3 (*p* < 0.05). Turkeys fed diets supplemented with synbiotics S1 and S3 had shorter small intestines than the remaining birds (*p* < 0.05). The weight of the small intestine was lower in turkeys fed diets with the addition of the probiotic preparation BioPlus 2B and synbiotic S1 (*p* < 0.05). The pH of small intestinal digesta was not affected by the analyzed additives. The length of the ceca tended to decrease in turkeys administered synbiotic S3 (0.05 < *p* < 0.10). The tested feed additives had no effect on the weight of the ceca in birds fed OTA-contaminated diets. The probiotic preparation of Cylactin and synbiotics S1 and S3 induced a significant decrease in the pH of cecal digesta (*p* < 0.05).

Gastrointestinal parameters in 15-week-old turkeys are presented in Table 5. The analyzed feed additives had no effect on the weight of the crop, proventriculus, or gizzard in turkeys fed OTA-contaminated diets. The pH of crop digesta was lower in birds fed diets supplemented with Cylactin than in the remaining groups (*p* < 0.05). Synbiotics S1 and S2 increased the pH of proventriculus digesta (*p* < 0.05) and decreased the pH of gizzard digesta (*p* < 0.05). Neither the length nor the pH of the small intestine was affected by the tested feed additives. The weight of the small intestine was lower (*p* < 0.05) in turkeys receiving synbiotics S1 and S2. The analyzed feed additives had no influence on the weight and length of the ceca in birds fed OTA-contaminated diets. The analyzed probiotics and synbiotics induced a decrease in the pH of cecal digesta, compared with the control group (*p* < 0.05).

### 3.3. The Carcass Quality 

The carcass quality characteristics of turkeys are presented in Table 6. At 15 weeks of age, the analyzed probiotics and synbiotics had no effect on the carcass dressing percentage or the proportions of breast muscles, abdominal fat, heart, and kidneys in birds fed OTA-contaminated diets. Turkeys receiving the probiotic preparation Cylactin and synbiotics S1 and S2 were characterized by a lower proportion of the liver in the carcass than the remaining birds (*p* < 0.001).

### 3.4. Concentration of Ochratoxin in Tissues, Organs, Digesta, and Feces

At 6 weeks of age, turkeys fed diets supplemented with feed additives were characterized by lower mycotoxin levels in the kidneys (*p* < 0.001). At 15 weeks of age, OTA levels were lower in birds administered synbiotics S1 and S2 (*p* < 0.001). (Table 7). At both 6 and 15 weeks of age, the accumulation of OTA was lower in the livers of turkeys receiving probiotics and synbiotics, relative to the control group (*p* < 0.001). The tested feed additives had no effect on OTA concentrations in the breast muscles of turkeys (Table 7).

Synbiotic S1 contributed to a decrease (*p* < 0.05) in serum OTA levels in 3-week-old turkeys (Table 8). Birds fed diets supplemented with synbiotics S1 and S2 had lower serum OTA levels in week 9 of the experiment. At 15 weeks of age, serum OTA levels decreased in turkeys receiving the probiotic preparation Cylactin and synbiotics (*p* < 0.01). 

The OTA content of jejunal digesta was highest in the control group and lowest in turkeys fed diets with the addition of synbiotics and the probiotic preparation BioPlus 2B in week 3 (*p* < 0.01), synbiotics and the probiotic preparation Cylactin in week 9 (*p* < 0.01), and synbiotics S1 and S3 and the probiotic preparation Cylactin in week 15 of the study (*p* < 0.05) (Table 9).

At 3 weeks of age, the OTA content of feces decreased significantly in turkeys fed diets supplemented with the probiotic preparation Cylactin and synbiotic S1, compared with the remaining groups (*p* < 0.05) (Table 9). In week 9 of the experiment, the OTA content of fecal samples was higher in birds administered the probiotic preparation BioPlus 2B and synbiotics S2 and S3 than in the control group (*p* < 0.05). In week 15 of the study, OTA concentration in feces was higher in turkeys administered synbiotic S2 and probiotic BioPlus 2B than in those receiving synbiotic S1 and control group birds (*p* < 0.05) (Table 9).

### 3.5. Health Indicators

At 3 weeks of age, serum lysozyme levels increased in turkeys fed diets supplemented with synbiotic S2, compared with the remaining groups (*p* < 0.01) (Tabel 10). Dietary supplementation with Cylactin and synbiotics induced a decrease in serum ceruloplasmin activity in turkeys exposed to OTA in feed (*p* < 0.01). Serum total protein was not affected by supplemental probiotics or synbiotics. Serum γ-globulin levels increased (by 27% on average) in birds administered synbiotic S3, relative to control group birds and those receiving synbiotics S1 and S2.

At 8 weeks of age, serum lysozyme levels increased significantly in turkeys fed diets with the addition of the probiotic preparation BioPlus 2B and synbiotics S1 and S2, in comparison with the remaining birds (*p* < 0.01) (Table 10). Turkeys fed diets supplemented with the probiotic preparation Cylactin and synbiotics S2 and S3 were characterized by increased ceruloplasmin activity (*p* < 0.01). Serum total protein levels were highest in birds receiving supplemental synbiotic S1 (*p* < 0.01). The tested feed additives had no influence on serum γ-globulin levels.

At 15 weeks of age, no differences in serum lysozyme and total protein levels were found in turkeys fed OTA-contaminated diets (Table 10). Dietary supplementation with synbiotics increased serum ceruloplasmin activity, relative to the remaining groups (*p* < 0.05). The probiotic preparation BioPlus 2B and synbiotic S1 added to OTA-contaminated diets contributed to increasing serum γ-globulin levels (*p* < 0.05).

## 4. Discussion

In the cultivation of food crops, protection against mycotoxins primarily involves preventive measures such as appropriate cultivation, harvesting, and storage conditions. The presence of OTA has been confirmed in various feed materials and livestock diets regardless of the country of origin [37]. The occurrence of contaminated feed components requires the use of different detoxification methods that should be safe for animals and economically viable. One of the proposed solutions to neutralize fungal toxins is the use of feed additives such as synbiotics, which combine prebiotic and probiotic properties, during feed production [38]. Bacterial and yeast strains are capable of biodegrading various mycotoxins [11,39,40,41].

In the present study, the addition of synbiotics S1 and S3 to OTA-contaminated diets contributed to an increase in the final body weight of turkeys at 6 weeks of age, compared with birds fed diets without additives or supplemented with probiotics. Similar results were noted by Markowiak et al. [13] and Tong et al. [42], who analyzed the efficacy of synbiotics containing lactic acid bacteria and *S. cerevisiae* yeasts and selenium-rich yeasts, respectively, in broiler chicken diets. However, in the current experiment, no improvement was observed in the final body weight of turkeys at day 105. Moreover, the average body weight of turkeys was lower than that published by the breeding company (7.70 vs. 10.45 kg) [43]. The detrimental effect of OTA on body weight gain, feed intake, and consequently, the FCR has been observed in several poultry species [44,45,46,47]. Their poorer growth performance was mainly due to reduced feed intake [48]. In poultry, OTA exerts nephrotoxic and hepatotoxic effects by inhibiting protein, DNA, and RNA synthesis and stimulating lipid peroxidation, which negatively affects the overall health status and growth performance of birds [49,50,51]. The efficacy of probiotics and synbiotics in neutralizing the adverse effects of OTA on bird productivity may vary depending on mycotoxin concentrations in contaminated diets. The fact that the tested feed additives did not improve the growth performance of turkeys in the described experiment could be due to the high OTA levels in the diets, which significantly exceeded the permitted limits. Similar observations have been made by other authors [45].

The gastrointestinal tract is where nutrients are digested and absorbed and is therefore directly exposed to mycotoxins present in feed. In this study, an increase in the weight and length of the small intestine was observed in turkeys fed OTA-contaminated diets, especially in young birds. Other authors have also observed histopathological changes in the intestines of birds, such as decreased size and atrophy of intestinal villi and increased size of crypts [52,53]. In the present study, the pH of cecal digesta decreased in turkeys fed diets supplemented with probiotics or synbiotics. The antagonistic effect of probiotics and synbiotics against pathogenic bacteria is due to the modification of intestinal pH, which occurs as a result of the production of volatile fatty acids and lactic acid. This process facilitates the exclusion of pH-sensitive pathogens, such as *Salmonella* and *E. coli* [54]. This mechanism of action helps maintain a balance between beneficial and pathogenic bacteria, which is essential for intestinal health and the proper growth of birds, especially those exposed to mycotoxins in feed. 

In the current experiment, synbiotics had no influence on the carcass dressing percentage, the proportions of individual organs in the carcass, or the chemical composition of breast muscles in turkeys fed OTA-contaminated diets. However, the proportion of the liver in the carcass decreased in birds fed OTA-contaminated diets supplemented with the probiotic preparation Cylactin and synbiotics 1 and 2. The toxic effects of OTA are manifested as swelling, congestion, and cell necrosis, probably due to the effort associated with eliminating toxins from the body, resulting in enlargement of the liver and other organs [49], as observed in numerous studies [55,56,57]. 

The presence of OTA in poultry diets decreases the serum levels of total protein, albumins, and globulins, probably due to reduced protein synthesis in the liver and damage to this organ [56]. In addition, OTA inhibits both humoral and cell-mediated immune responses in animals [58]. Lysozyme is an antibacterial enzyme that exerts immunostimulatory effects [59]. Ceruloplasmin is an acute-phase protein enzyme of the multicopper oxidase family, known for its antioxidant properties [60]. Chronic exposure to toxic factors such as mycotoxins in feed decreases the serum levels of lysozyme and ceruloplasmin [61,62]. In the present study, turkeys fed OTA-contaminated diets were characterized by lower serum levels of total protein and γ-globulins at different life stages, which corroborates the findings of other researchers [44,62], and these undesirable changes were attenuated by probiotic and synbiotic supplements (increased lysozyme and ceruloplasmin activity). Similar results were reported by other authors who found that blood biochemical parameters improved in birds fed mycotoxin-contaminated diets supplemented with probiotics [63,64]. Research has also shown that probiotics and synbiotics enhance humoral immune responses [65]. 

Ochratoxins tend to accumulate in the tissues and organs of animals exposed to their presence in feed [49], and OTA is deposited in the tissues of birds regardless of its concentration in the diet [62,66]. The ability of OTA to bind to serum proteins, mainly albumins, contributes to its distribution throughout the body [67]. At the European Union level, Commission Regulation (EC) No. 2023/915 [68] sets maximum levels for OTA in various foodstuffs, but no limits have been set for meat and other foods of animal origin. However, in Poland and some other countries, poultry meat products are subject to guidelines limiting exposure to OTA to the maximum level of 5.0 μg/kg [69]. In the current study, probiotic and synbiotic feed additives had no effect on OTA concentrations in the breast muscles of turkeys fed OTA-contaminated diets (0.92 µg/kg on average); however, these levels were below the permissible exposure limit in Poland (5.0 µg/kg). The OTA content of the kidneys, liver, blood, and jejunal digesta was lower in turkeys fed OTA-contaminated diets supplemented with probiotics or synbiotics. The increased OTA content of fecal samples indicates that synbiotics S1 and S2 and the probiotic preparation BioPlus 2B are able to bind to mycotoxins and eliminate them from the body. These results are consistent with the findings of other authors who added bacterial and yeast strains to poultry diets [53,66,70]. *Saccharomyces cerevisiae* yeasts are able to remove mycotoxins from the body due to the presence of oligomannans in the structure of their cell walls, which bind to toxins [71]. Lactic acid bacteria remove mycotoxins by adhering to their cell walls [72]. They are also capable of metabolizing OTA to nontoxic forms such as ochratoxin alfa (Otα) [73]. Research has demonstrated that the inclusion of feed additives containing lactic acid bacteria and *S. cerevisiae* yeast strains in poultry diets can reduce the accumulation of OTA in the birds’ internal organs.

The feed additives, evaluated under conditions where the permissible level of ochratoxin in the feed mixtures was exceeded by several factors, demonstrated effectiveness in improving animal health (increased lysozyme and ceruloplasmin activity) and reducing toxin accumulation in the internal organs of turkeys.

## 5. Conclusions

The addition of probiotic and synbiotic preparations based on lactic acid bacteria strains, inulin, and *S. cerevisiae* yeasts to OTA-contaminated diets of turkeys in commercial farms may improve their health status and reduce mycotoxin accumulation in the organs and tissues of poultry.

## Figures and Tables

**Table 1:** The composition and nutritional value of turkey complete diets.

Item	Starter 1	Starter 2	Grower 1	Grower 2	Finisher
	0–3 Weeks	4–6 Weeks	7–9 Weeks	10–12 Weeks	13–15 Weeks
Ingredient [g/kg]					
Wheat	261.9	310.3	416.7	515.8	590.4
Corn	200.0	200.0	150.0	100.0	100.0
Soybean meal	358.2	360.9	347.4	300.0	225.1
Full-fat soybeans	100.0	50.0	-	-	-
Blood meal	20.0	10.0	-	-	-
Soybean oil	5.2	19.2	39.1	45.1	47.8
L-lysine HCl	3.1	3.6	3.7	3.2	4.0
DL-methionine	3.5	2.6	2.4	2.6	2.5
L-threonine	0.7	0.7	1.1	0.7	1.0
Limestone	18.8	14.5	13.4	11.1	9.7
Monocalcium phosphate	22.1	19.9	17.7	13.1	11.1
Sodium bicarbonate	0.1	1.3	1.2	0.7	0.7
NaCl	2.0	1.9	2.2	2.6	2.7
Feed enzymes	0.1	0.1	0.1	0.1	0.1
Premix *	5.0	5.0	5.0	5.0	5.0
Nutritional value of feed					
ME, [kcal/kg]	2800	2880	3000	3100	3200
CP, g	27.51	25.03	23.69	22.98	21.18
Lys, [%]	1.77	1.65	1.45	1.30	1.17
Met+Cys, [%]	1.15	1.02	0.95	0.93	0.85
Ca, [g]	1.40	1.20	1.15	1.00	0.90
Available P, [g]	0.70	0.65	0.60	0.50	0.45
Na, [g]	0.13	0.15	0.15	0.15	0.15
Ochratoxin A (µg/kg)	198.6	251.6	331.0	397.2	462.0

* Composition of the premix for Starter 1 and 2 diets—12,500 IU vitamin A, 4500 IU vitamin D3, 87.5 mg vitamin E, 3.75 mg vitamin K3, 3.5 mg vitamin B1, 10 mg vitamin B2, 75 mg niacin, 22.5 mg pantothenic acid, 6.0 mg vitamin B6, 30 µg vitamin B12, 2.5 mg folic acid, 400 µg biotin, 800 mg choline chloride, 92.5 mg Fe, 130 mg Mn, 20 mg Cu, 105 mg Zn, 2.5 mg I, and 0.3 mg Se; Grower 1 and 2 diets—11,500 IU vitamin A, 4140 IU vitamin D3, 80.5 mg vitamin E, 3.45 mg vitamin K3, 3.22 mg vitamin B1, 9.2 mg vitamin B2, 69 mg niacin, 20.7 mg pantothenic acid, 5.52 mg vitamin B6, 37.6 µg vitamin B12, 2.3 mg folic acid, 368 µg biotin, 600 mg choline chloride, 85.1 mg Fe, 120 mg Mn, 18.4 mg Cu, 96.6 mg Zn, 2.3 mg I, and 0.26 mg Se; Finisher diet—10,500 IU vitamin A, 3780 IU vitamin D3, 66.5 mg vitamin E, 2.85 mg vitamin K3, 2.66 mg vitamin B1, 7.6 mg vitamin B2, 57 mg niacin, 17.1 mg pantothenic acid, 4.6 mg vitamin B6, 22.8 µg vitamin B12, 1.9 mg folic acid, 304 µg biotin, 400 mg choline chloride, 70.3 mg Fe, 98.8 mg Mn, 15.2 mg Cu, 79.8 mg Zn, 1.9 mg I, and 0.23 mg Se.

**Table 2:** Experimental design.

Groups	Number of Turkeys	OTA in Feed Mixture	Feed Additive	Composition of the Feed Additive
OTA	20	Present	no additive	N/A
BioPlus 2B	20	Present	BioPlus 2B	*B. licheniformis DSM 5749* *B. subtilis DSM 5750*
Cylactin	20	Present	Cylactin	*E. faecium NCIMB 10415* *(SF68)*
S1	20	Present	Synbiotic 1	*Lb. plantarum ŁOCK 086* *Lb. reuteri ŁOCK 1092* *Lb. pentosus ŁOCK 1094* *S. cerevisiae ŁOCK 0119* inulin
S2	20	Present	Synbiotic 2	*Lb. plantarum ŁOCK 0860* *Lb. reuteri ŁOCK 1092* *Lb. pentosus ŁOCK 1094* *Lb. rhamnosus ŁOCK 1087* *S. cerevisiae ŁOCK 0119* inulin
S3	20	Present	Synbiotic 3	*Lb. plantarum ŁOCK 0860* *Lb. reuteri ŁOCK 1092* *Lb. pentosus ŁOCK 1094* *Lb. rhamnosus ŁOCK 1087* *Lb. paracasei ŁOCK 1091* *S. cerevisiae ŁOCK 0119* inulin

**Table 3:** Growth performance of turkeys in the 6th and 15th weeks of age.

Item	Groups						SEM	*p*
	Ochratoxin	BioPlus 2B	Cylactin	S1	S2	S3		
Age of birds—Week 6								
BW †, kg per bird	1.65 ^b^	1.64 ^b^	1.65 ^b^	1.78 ^a^	1.67 ^b^	1.78 ^a^	0.015	0.017
Cumulative Feed Intake, kg per bird	3.49	3.54	3.58	3.65	3.58	3.80	0.027	0.122
FCR ‡, kg/kg	2.31	2.36	2.37	2.23	2.34	2.32	0.018	0.255
Age of birds—Week 15								
BW †, kg per bird	7.60	7.63	7.79	7.80	7.63	7.72	0.045	0.668
Cumulative feed intake, kg per bird	20.81	20.90	21.42	20.76	20.97	21.22	0.015	0.487
FCR, kg/kg	2.79	2.79	2.80	2.71	2.80	2.80	0.043	0.369

^a,b^—values in rows with different letters are significantly different for *p* < 0.05); † BW, body weight; ‡ FCR, feed conversion ratio.

**Table 4:** The structure of the gastrointestinal tract and digesta pH in 6-week-old turkeys.

	Groups							
Item	Ochratoxin	BioPlus 2B	Cylactin	S1	S2	S3	SEM	*p*
Crop								
- weight, g/kg BW.	4.71	4.86	4.41	4.56	4.61	4.02	0.205	0.171
- pH of digesta	4.85 ^b^	4.88 ^b^	4.87 ^b^	5.25 ^b^	6.21 ^a^	4.82 ^b^	0.098	0.001
Proventriculus								
- weight, g/kg BW.	3.68	3.50	3.80	3.51	3.58	3.50	0.048	0.391
- pH of digesta	3.38 ^ab^	3.91 ^a^	3.22 ^b^	3.62 ^ab^	3.80 ^a^	3.92 ^a^	0.063	0.001
Gizzard								
- weight, g/kg BW.	22.74	22.42	22.90	22.59	23.11	21.89	0.411	0.978
- pH of digesta	2.58	2.77	2.48	2.34	2.30	2.52	0.066	0.351
Small intestine								
- length, cm/kg BW	103.91 ^ab^	107.26 ^a^	103.32 ^ab^	91.23 ^b^	103.30 ^ab^	92.34 ^b^	1.841	0.047
- weight, g/kg BW.	30.17 ^ab^	25.74 ^c^	28.47 ^abc^	26.43 ^bc^	31.26 ^a^	29.12 ^abc^	0.470	0.001
- pH of digesta	6.27	6.14	6.10	6.46	6.33	6.44	0.050	0.214
Ceca								
- length, cm/kg BW	31.55 ^x^	32.29 ^x^	29.77 ^xyz^	29.42 ^xyz^	31.93 ^xy^	27.78 ^z^	0.484	0.054
- weight, g/kg BW.	5.53	5.63	5.55	5.16	5.13	5.48	0.082	0.326
- pH of digesta	7.11 ^a^	6.79 ^ab^	6.49 ^b^	6.60 ^b^	6.71 ^ab^	6.47 ^b^	0.062	0.016

^a,b,c^—values in rows with different letters are significantly different for *p* < 0.05; ^x,y,z^ comparison shows a trend for 0.05 < *p* < 0.10.

**Table 5:** The structure of the gastrointestinal tract and digesta pH in 15-week-old turkeys.

	Groups							
Item	Ochratoxin	BioPlus 2B	Cylactin	S1	S2	S3	SEM	*p*
Crop								
- weight, g/kg BW.	3.28	3.54	3.56	3.06	2.79	3.17	0.104	0.245
- pH of digesta	5.25 ^ab^	4.94 ^bc^	4.85 ^c^	5.08 ^abc^	5.31 ^a^	4.97 ^bc^	0.046	0.017
Proventriculus								
- weight, g/kg BW.	1.44	1.40	1.45	1.34	1.37	1.35	0.018	0.366
- pH of digesta	3.28 ^ab^	3.14 ^b^	3.61 ^ab^	3.70 ^a^	3.72 ^a^	3.17 ^b^	0.071	0.024
Gizzard								
- weight, g/kg BW.	10.86	11.11	11.00	10.87	12.03	11.91	0.196	0.283
- pH of digesta	3.16 ^ab^	3.46 ^a^	3.09 ^ab^	2.78 ^b^	2.90 ^b^	3.05 ^ab^	0.063	0.033
Small intestine								
- length, cm/kg BW	30.40	31.17	30.16	28.09	30.65	30.77	0.344	0.120
- weight, g/kg BW.	13.86 ^ab^	13.02 ^ab^	14.21 ^a^	11.58 ^c^	12.24 ^bc^	13.06 ^ab^	0.216	0.002
- pH of digesta	6.49	6.41	6.50	6.37	6.32	6.47	0.031	0.462
Ceca								
- length, cm/kg BW	10.06	10.68	9.69	9.98	9.99	9.79	0.116	0.124
- weight, g/kg BW.	3.09	3.10	3.09	2.85	2.88	3.00	0.046	0.432
- pH of digesta	7.22 ^a^	6.63 ^b^	6.58 ^b^	6.71 ^b^	6.63 ^b^	6.71 ^b^	0.056	0.006

^a,b,c^—values in rows with different letters are significantly different for *p* < 0.05.

**Table 6:** Carcass quality characteristics in 15-week-old turkeys (% per bird).

	Groups							
Item	Ochratoxin	BioPlus 2B	Cylactin	S1	S2	S3	SEM	*p*
Carcass dressing percentage (%)	77.93	77.68	78.84	78.49	78.58	78.34	0.788	0.988
Breast muscle content of carcass (%)	26.88	26.35	26.65	27.26	26.45	26.44	0.244	0.890
Abdominal fat content relative to BW (%)	1.40	1.43	1.39	1.39	1.51	1.55	0.025	0.266
Heart content of carcass (%)	0.39	0.40	0.38	0.40	0.43	0.45	0.007	0.702
Liver content of carcass (%)	2.03 ^a^	1.84 ^a^	1.62 ^b^	1.56 ^b^	1.57 ^b^	1.81 ^ab^	0.040	<0.001
Kidney content of carcass (%)	0.36	0.35	0.28	0.36	0.40	0.34	0.020	0.632

^a,b^—values in rows with different letters are significantly different for *p* < 0.05.

**Table 7:** Concentrations of ochratoxin A in the breast muscles, kidneys, and liver of turkeys.

	Groups							
Item	Ochratoxin	BioPlus 2B	Cylactin	S1	S2	S3	SEM	*p*
Kidneys (µg/kg)								
Age of birds, weeks								
- 6	5.78 ^a^	4.46 ^b^	3.67 ^b^	2.93 ^b^	3.62 ^b^	3.66 ^b^	0.185	<0.001
- 15	4.54 ^a^	3.82 ^ab^	4.50 ^a^	3.17 ^b^	3.23 ^b^	3.52 ^ab^	0.162	0.030
Liver (µg/kg)								
Age of birds, weeks								
- 6	4.51 ^a^	3.60 ^b^	3.52 ^b^	3.40 ^b^	2.46 ^c^	2.95 ^b^	0.145	<0.001
- 15	3.80 ^a^	2.36 ^b^	2.83 ^b^	2.16 ^b^	2.14 ^b^	2.74 ^b^	0.121	<0.001
Breast muscles (µg/kg)								
Age of birds, weeks								
- 6	0.92	0.87	0.86	0.78	0.77	0.79	0.029	0.623
- 15	0.96	0.91	0.94	0.93	0.92	0.89	0.019	0.937

^a,b,c^—values in rows with different letters are significantly different for *p* < 0.05.

**Table 8:** Blood ochratoxin A levels in turkeys.

	Groups							
Item	Ochratoxin	BioPlus 2B	Cylactin	S1	S2	S3	SEM	*p*
Blood (µg/kg)								
Age of birds, weeks								
- 3	18.61 ^a^	18.25 ^a^	16.49 ^ab^	13.75 ^b^	19.35 ^a^	15.93 ^ab^	0.566	0.013
- 9	12.94 ^a^	10.89 ^ab^	10.54 ^ab^	6.77 ^c^	8.62 ^bc^	10.29 ^abc^	0.617	0.047
- 15	12.73 ^a^	9.61 ^ab^	5.59 ^b^	5.74 ^b^	5.56 ^b^	7.06 ^b^	0.773	0.008

^a,b,c^—values in rows with different letters are significantly different for *p* < 0.05.

**Table 9:** Concentrations of ochratoxin A in the jejunal digesta and feces of turkeys.

	Groups							
Item	Ochratoxin	BioPlus 2B	Cylactin	S1	S2	S3	SEM	*p*
Jejunal digesta (µg/kg)								
Age of birds, weeks								
- 3	151.82 ^a^	102.81 ^b^	126.07 ^ab^	57.04 ^c^	105.97 ^b^	106.83 ^b^	5.771	<0.001
- 9	177.62 ^a^	163.31 ^ab^	116.66 ^bc^	97.40 ^c^	96.21 ^c^	147.26 ^b^	6.609	<0.001
- 15	73.34 ^a^	60.16 ^ab^	40.26 ^b^	34.63 ^b^	54.93 ^ab^	45.98 ^b^	3.881	0.033
Feces (µg/kg)								
Age of birds, weeks								
- 3	20.62 ^a^	17.49 ^ab^	6.33 ^c^	9.54 ^bc^	15.01 ^abc^	16.15 ^abc^	1.433	0.015
- 9	25.00 ^b^	53.50 ^a^	43.24 ^ab^	39.10 ^ab^	49.53 ^a^	51.76 ^a^	2.897	0.025
- 15	62.98 ^b^	75.76 ^a^	67.27 ^ab^	62.08 ^b^	90.20 ^a^	67.22 ^ab^	2.736	0.031

^a,b,c^—values in rows with different letters are significantly different for *p* < 0.05.

**Table 10:** Activity of ceruloplasmin and lysozyme and serum total protein and γ-globulin levels in turkeys.

	Groups							
Item	Ochratoxin	BioPlus 2B	Cylactin	S1	S2	S3	SEM	*p*
Week 3								
Lysozyme, mg/L	7.64 ^b^	10.44 ^ab^	6.58 ^b^	8.82 ^b^	13.50 ^a^	8.34 ^b^	0.546	0.002
Ceruloplasmin, IU	51.33 ^a^	51.16 ^a^	46.61 ^b^	47.74 ^b^	47.62 ^b^	46.84 ^b^	0.452	0.001
Total protein, g/L	37.36	38.05	37.39	37.84	36.27	37.00	0.260	0.431
γ-globulins, g/L	4.90 ^b^	5.73 ^ab^	5.80 ^ab^	5.06 ^b^	4.80 ^b^	6.28 ^a^	0.154	0.026
Week 9								
Lysozyme, mg/L	14.41 ^c^	38.33 ^a^	17.41 ^c^	30.63 ^b^	26.75 ^b^	18.27 ^c^	1.346	<0.001
Ceruloplasmin, IU	42.25 ^b^	43.29 ^ab^	44.25 ^a^	41.74 ^bc^	43.47 ^a^	43.18 ^a^	0.205	0.003
Total protein, g/L	39.06 ^bc^	38.71 ^bc^	40.19 ^ab^	40.87 ^a^	38.37 ^bc^	38.71 ^bc^	0.220	0.002
γ-globulins, g/L	6.52	6.62	7.08	6.83	5.98	6.52	0.179	0.624
Week 15								
Lysozyme, mg/L	6.80	10.94	7.38	7.80	8.20	9.24	0.606	0.420
Ceruloplasmin, IU	46.63 ^b^	45.94 ^c^	47.77 ^abc^	48.87 ^a^	48.17 ^a^	48.95 ^a^	0.312	0.020
Total protein, g/L	40.82	45.56	41.95	46.38	43.40	43.85	0.655	0.120
γ-globulins, g/L	7.25 ^b^	12.38 ^a^	6.71 ^b^	12.32 ^a^	9.21 ^ab^	8.30 ^ab^	0.641	0.023

^a,b,c^—values in rows with different letters are significantly different for *p* < 0.05.

## Data Availability

The data presented in this study are available from the corresponding author.
